# Sleep Lab Adaptation in Children with Attention-Deficit/Hyperactivity Disorder and Typically Developing Children

**DOI:** 10.1155/2013/698957

**Published:** 2013-05-12

**Authors:** Meredith Bessey, Jennifer Richards, Penny Corkum

**Affiliations:** ^1^Department of Psychology & Neuroscience, Dalhousie University, P.O. Box 15000, Halifax, NS, Canada B3H 4R2; ^2^IWK Health Centre, 5850 University Avenue, Halifax, NS, Canada B3K 6R8; ^3^Colchester East Hants ADHD Clinic, 600 Abenaki Road, Truro, NS, Canada B2N 5A1

## Abstract

*Objectives*. Research has shown inconsistencies across studies examining sleep problems in children with attention-deficit/hyperactivity disorder (ADHD). It is possible that these inconsistencies are due to sleep lab adaptation. The goal of the current study was to investigate the possibility that children with ADHD adapt differently to the sleep lab than do typically developing (TD) children. *Patients and Methods*. Actigraphy variables were compared between home and the sleep lab. Sleep lab adaptation reports from the parent and child were compared between children with ADHD (*n* = 25) and TD children (*n* = 25). *Results*. Based on actigraphy, both groups had reduced sleep duration and reduced wake after sleep onset in the sleep lab compared to home. The only interaction effect was that TD children had increased sleep efficiency in the sleep lab compared to home. *Conclusions*. The results of this study do not support the hypothesis that children with ADHD adjust to the sleep lab differently than their typically developing peers. However, both groups of children did sleep differently in the sleep lab compared to home, and this needs to be considered when generalizing research findings from a sleep lab environment to children's sleep in general.

## 1. Introduction

There have been growing research and clinical interest in the relationship between attention-deficit/hyperactivity disorder (ADHD) and sleep. Parent reports have consistently reported high rates of sleep problems in children with ADHD [1]; however, results from polysomnography (PSG) are inconsistent. There are several possibilities for these inconsistencies; for example, studies have often failed to match ADHD and typically developing (TD) groups on age and sex and lack control for stimulant medication use [2]. Another possibility is that sleep lab adaptation may be different for children with ADHD. The sleep lab is an unfamiliar environment that involves interacting with new people and sleeping with electrodes. This could lead to atypical sleep, helping to explain previous research inconsistencies.

 The goal of the current study was to investigate sleep lab adaptation in children with ADHD and TD children, using actigraphy and parent- and child-completed questionnaires. It is hypothesized (a) that both groups of children will be negatively affected by the sleep lab environment, with children with ADHD possibly being more negatively affected, and (b) that parent and child reports will indicate that children with ADHD have more difficulty sleeping in the sleep lab than TD children. 

## 2. Materials and Methods

### 2.1. Participants

A total of 50 children (25 with ADHD and 25 TD children), ages 6 to 12, participated in the study. All children with ADHD were recently rigorously diagnosed and medication naive. None of the participants had previous sleep lab experience or had comorbid, primary mental health, sleep, neurological, or genetic disorders.

### 2.2. Measures

#### 2.2.1. Conners' Rating Scale-Revised Long Version (CRS-R:L [3])

Both the Conners' Parent (CPRS) and Teacher (CTRS) were used as screening tools. The Conners' ADHD index was the only subscale analyzed. 

#### 2.2.2. Demographic Questionnaire

This questionnaire was used to compare both groups on possible confounding variables [4]. 

#### 2.2.3. Actigraphy

Mini-Motionlogger Actigraphs (Ambulatory Monitoring Inc.) were used to collect data on four variables: (1) sleep duration, (2) sleep onset latency, (3) wake after sleep onset (WASO), and (4) sleep efficiency.

#### 2.2.4. Sleep Lab Adaptation Questionnaire (SLAQ)

This investigator-developed measure includes five questions for parents and children. Questions focus on sleep onset, sleep duration, general sleep quality, overall sleep lab experience, and how child friendly the sleep lab appeared. 

### 2.3. Procedure

The study received ethical approval from the research ethics board at the hospital and ADHD clinic. First the child wore the actigraph for six nights. The parent and teacher completed the CTRS and CPRS. Then the overnight PSG study took place. Upon arrival at the sleep lab, the research assistant (RA) played a game with the child and the technician conducted the PSG hook-up, which took approximately one hour.

Once the hook up was complete, the child followed their usual weekday bedtime routine and an actigraph was placed on the child's wrist before bedtime. A technician monitored the child's sleep overnight. The following morning, upon waking at their usual weekday wake time, the electrodes and actigraph were removed from the child, and the parent and child completed the SLAQ. (The SLAQ was added to the study protocol following the beginning of this study, and therefore some SLAQ data was collected at a later time point for some of the participants.) 

## 3. Results and Discussion

### 3.1. Sample Characteristics

The two groups (TD and ADHD) did not differ on mean age (*t*(48) = 0.10, *P* = .920; ADHD = 106.10 months (SD = 22.47 months), TD children = 105.47 months (SD = 23.00 months)), sex (*P* = 1.00; 22 boys, 3 girls in each group), parental marital status (*P* = 1.000), SES (*t*(48) = −0.75, *P* = .46), or number of children in the home (*F*(1, 48) = 1.438, *P* = .24). Expectedly, the two groups differed significantly on the mean ADHD index *t*-score for the CTRS, *t*(38) = 6.45, *P* < .001 (ADHD = 68.54 (SD = 9.75), TD = 48.94 (SD = 7.95)), and the CPRS, *t*(47) = 10.23, *P* < .001 (ADHD = 70.36 (SD = 9.69), TD = 46.00 (SD = 6.57)).

### 3.2. Hypothesis 1

Sleep latency of children with ADHD was 31.66 minutes (SD = 24.43) at home, versus 32.00 minutes (SD = 29.79) at the sleep lab. For TD children, latency was 22.05 minutes (SD = 16.82) at home, versus 24.28 minutes (SD = 18.10) in the sleep lab; neither the main effect (*F*(1, 48) = 0.08, *P* = .78) nor interaction (*F*(1, 48) = 0.04, *P* = .84) was significant. At home, sleep efficiency of children with ADHD was 83.35% (SD = 10.42%) and in the sleep lab 83.42% (SD = 10.01%). In TD children, sleep efficiency was 79.90% (SD = 10.76%) at home and 86.11% (SD = 7.25%) in the sleep lab; the main effect (*F*(1, 48) = 5.79, *P* = .020) and the interaction were significant (*F*(1, 48) = 5.53, *P* = .023). Sleep duration for children with ADHD was 571.03 minutes (SD = 42.23) at home versus 513.44 minutes (SD = 72.14) in the sleep lab. In TD children, sleep duration at home was 562.25 minutes (SD = 47.63) versus 525.84 minutes (SD = 62.58) in the sleep lab; the main effect was significant (*F*(1, 48) = 21.34, *P* < .001), but not the interaction (*F*(1, 48) = 1.08, *P* = .30). In children with ADHD, WASO was 95.27 minutes (SD = 57.80) at home and 86.08 minutes (SD = 55.50) in the sleep lab. In TD children, WASO was 111.47 minutes (SD = 58.31) at home and 73.52 minutes (SD = 41.28) in the sleep lab; again, the main effect was significant (*F*(1, 48) = 10.03, *P* = .003) but not the interaction (*F*(1, 48) = 3.74, *P* = .059).

### 3.3. Hypothesis 2

Items on the SLAQ indicated no differences in sleep lab adaptation between children with ADHD and TD children (Table 1). Although the parents and children in both groups reported a positive experience in the sleep lab, they also reported that sleep was slightly worse than what was normal at home.

### 3.4. Discussion

The goal of this study was to examine sleep lab adaptation in children with ADHD compared to TD children. Our results indicate that both groups of children slept approximately 50 minutes less while in the sleep lab and that both groups of children had fewer wake episodes in the sleep lab than at home. Interestingly, sleep efficiency of TD children improved by approximately 6% in the sleep lab, with no improvement in children with ADHD. Parents and children did not report significant differences between the two groups in terms of how the children adapted to sleeping in the sleep lab environment, and overall both groups of children were reported to have had a positive experience in the sleep lab, with sleep being slightly worse than sleep at home. 

Based on our hypothesis that all children would be negatively affected by being in the sleep lab environment, the finding of reduced sleep duration in both groups is not surprising. The sleep lab is a novel environment that could be intimidating to the child, leading to shortened sleep. Therefore, generalization of data about sleep duration based on studies using PSG in a sleep lab should be interpreted with caution, as the data may not accurately represent the child's typical sleep. It is important that clinicians use judgement when using PSG parameters to make recommendations to their patients about sleep.

The finding of reduced WASO in the sleep lab was not consistent with our hypothesis of generally poorer sleep in the sleep lab. As discussed above, the sleep lab is a highly structured environment, with no siblings, pets, and so forth to disrupt the child's sleep. This may have reduced night waking in all children, regardless of whether the child has ADHD or not. Another possibility is that sleep efficiency improved given the shorter sleep duration.

The finding of improved sleep efficiency in TD children, but not in children with ADHD, was surprising. It suggests that perhaps the sleep lab is conducive to better sleep in TD children but not in children with ADHD, at least in terms of some sleep variables. The sleep lab tends to be a more organized and structured environment than the home environment, as there are no distractions (e.g., siblings, and noises from other rooms) to disrupt the child's sleep. Perhaps this highly structured environment promoted better sleep efficiency in TD children; however, it is unclear why children with ADHD did not benefit. It is possible that rather than being more negatively affected by the sleep lab, as hypothesized, children with ADHD are less able than their typically developing peers to benefit from the potentially sleep-promoting effects of the sleep lab. 

## 4. Conclusion

Overall, this study provides interesting findings that are useful both clinically and in a research setting. Findings should be cautiously generalized from PSG to the home, as our results indicate that all children are affected by the sleep lab, with some sleep variables being negatively affected (i.e., sleep duration) and some being positively affected (i.e., WASO). In opposition to our hypothesis, it was found that children with ADHD were not differentially affected based on most variables analyzed in this study. Further research could investigate whether these findings extend to at-home PSG assessment. It is important to note that this study only examined variables that could be assessed by both actigraphy and PSG, and therefore it is not possible to comment on how the sleep lab may affect sleep architecture or sleep movements. As the data for this study was drawn from a child's first night in the sleep lab, it would also be of interest to examine sleep lab adaptation over a number of nights in the sleep lab, during which the children would be expected to habituate to the environment.

Overall, it can be concluded that because the sleep of children with ADHD was not differentially affected by the sleep lab experience, it is unlikely that the sleep lab contributes to the inconsistencies in the literature regarding sleep problems in children with ADHD. Given that children with ADHD did not differ significantly from TD children in terms of adaptation to the sleep lab, it is likely that the differences we see in research are due to the heterogeneity of ADHD in terms of symptom presentation (e.g., hyperactivity, attention), comorbidities, and medication. 

## Figures and Tables

**Table 1:** Mean values on parent and child SLAQ.

Variable	ADHD (SD)	TD (SD)	*P* value
SLAQ Parent			
Do you think the PSG hook-up (e.g., wires, finger clip, chest bands) changed how long it took your child to *fall* asleep compared to his/her sleep at home?	2.44 (0.82)	2.64 (0.81)	.39
Do you think the PSG hook-up changed how long your child *slept for compared to home*?	2.28 (0.74)	2.48 (0.65)	.32
How was the Sleep Lab bedroom?	4.32 (0.75)	4.44 (0.82)	.59
How was your child's sleep in the Sleep Lab?	3.32 (0.90)	3.38 (0.78)	.80
Rate your child's over-all experience in the Sleep Lab.	4.52 (0.71)	4.44 (0.65)	.68
SLAQ Child			
Do you think the PSG hook-up (e.g., wires, finger clip, chest bands) changed how long it took you to *fall* asleep compared to your sleep at home?	2.16 (0.80)	2.70 (1.15)	.06
Do you think the PSG hook-up changed how long you *slept for compared to home*?	2.52 (0.83)	2.64 (1.04)	.65
How did you like the Sleep Lab bedroom?	4.32 (0.75)	4.28 (0.79)	.86
How was your sleep in the Sleep Lab?	3.56 (1.00)	3.92 (1.15)	.24
Rate your over-all experience in the Sleep Lab?	4.20 (0.96)	4.16 (0.99)	.88

Note: The SLAQ items were scored on a 1–5 scale, with 1 signifying a worse than typical sleep, 3 signifying no change and 5 signifying a better than typical sleep.
